# Divergence of Acoustic Signals in a Widely Distributed Frog: Relevance of Inter-Male Interactions

**DOI:** 10.1371/journal.pone.0087732

**Published:** 2014-01-28

**Authors:** Nelson A. Velásquez, Daniel Opazo, Javier Díaz, Mario Penna

**Affiliations:** 1 Programa de Fisiología y Biofísica, ICBM, Facultad de Medicina, Universidad de Chile, Independencia, Santiago, Chile; 2 Laboratorio de Neurobiología y Biología del Conocer, Departamento de Biología, Facultad de Ciencias, Universidad de Chile, Las Palmeras, Santiago, Chile; Universite Paris XI – CNRS, France

## Abstract

Divergence of acoustic signals in a geographic scale results from diverse evolutionary forces acting in parallel and affecting directly inter-male vocal interactions among disjunct populations. *Pleurodema thaul* is a frog having an extensive latitudinal distribution in Chile along which males' advertisement calls exhibit an important variation. Using the playback paradigm we studied the evoked vocal responses of males of three populations of *P. thaul* in Chile, from northern, central and southern distribution. In each population, males were stimulated with standard synthetic calls having the acoustic structure of local and foreign populations. Males of both northern and central populations displayed strong vocal responses when were confronted with the synthetic call of their own populations, giving weaker responses to the call of the southern population. The southern population gave stronger responses to calls of the northern population than to the local call. Furthermore, males in all populations were stimulated with synthetic calls for which the dominant frequency, pulse rate and modulation depth were varied parametrically. Individuals from the northern and central populations gave lower responses to a synthetic call devoid of amplitude modulation relative to stimuli containing modulation depths between 30–100%, whereas the southern population responded similarly to all stimuli in this series. Geographic variation in the evoked vocal responses of males of *P. thaul* underlines the importance of inter-male interactions in driving the divergence of the acoustic traits and contributes evidence for a role of intra-sexual selection in the evolution of the sound communication system of this anuran.

## Introduction

Animal communication signals convey information from senders to receivers contributing prezygotic barriers, promoting by these means reproductive isolation [1] and speciation [2]. However, signals have been shown to exhibit important intra-specific variation related to geographical distribution. Such differences among populations have been related to environmental factors [3] [4] [5] and to evolutionary divergence [2] [6] [7] [8] [9].

As originally proposed by Darwin, conspicuous signals mediate female's choice of particular males (inter-sexual selection) and competition among males for access to females (intra-sexual selection) [10] [11] [12]. Geographic variation of signals can have strong effects on both mate choice and inter-male interactions, because small variations in the signal structure may alter significantly the outcome of these behaviors [13] [14] [15] [16] [17]. The study of mate choice has received considerable attention from behavioral biologists and its importance for the evolution of communication signals has been extensively acknowledged. These efforts have encompassed studies on the influence of geographic variation of signals on female preferences [18] [19] [20] and on signal evolution [21] [22] [23]. In contrast with studies focusing on the relevance of signal variation for female behavior, the significance of geographic variation of vocalizations for signal recognition among vocal-interacting males has received limited attention.

Anurans use acoustic communication extensively, this being their main channel for information exchange. During the breeding season, males emit advertisement calls that attract females and signal territories to other males. Geographic variation of these acoustic signals has been widely studied [24] [25] [26] [27] [19] [28] [29] and diverse evolutionary forces have been considered to play a role in this divergence. For instance, evolution of bioacoustic traits may follow phylogenetic divergence, in which case genetic drift has been proposed as the most parsimonious explanation for diverging populations [30] [31]. In addition it has been shown that ecological selection has played a role in signal divergence. Males of the subspecies *Acris c. crepitans*, an inhabitant from North American eastern forest habitats, produce calls having higher frequencies and faster pulse rates that are not degraded in their native environments to the same extent as calls of the subspecies *Acris c. blanchardi*, from western open habitats, which have slower pulse rates and lower frequencies [32] [24]. Furthermore, females of two species of the genus *Engystomops (E. pustulosus and E. petersi)* prefer local calls to calls from foreign populations, a preference that highlights the importance of inter-sexual selection for trait divergence [19] [27]. However, the relevance of geographic variation for the recognition of acoustic signals as measured by male evoked vocal responses in a framework of intra-sexual selection acting on signal evolution has received virtually no attention.

*Pleurodema thaul* (Leiuperidae) is an anuran with a wide geographical distribution in Chile, ranging from the Atacama desert (27° 06′ S, 69° 53′ W [33]) to the Patagonian region (45° 24′ S, 72° 42′ W [34] [33]). During the breeding season (from May to December, depending on the locality), males congregate in chorusing assemblages. A recent study has shown that males emit advertisement calls differing among populations along their distribution in both temporal and spectral structure, and these differences are highly correlated with genetic variation [29]. The differences at both the acoustic and genetic level revealed the existence of three bioacoustic groups, in correspondence with the lineages previously proposed for *P. thaul* in Chile [35] [36]. A recent review by Wilkins et al. (2013) has put forward the notion that correlations between bioacoustic and genetic divergence may not preclude the implication of factors other than genetic drift in signal evolution [37].

A first goal of the current study is to investigate the role of intra-sexual selection as an evolutionary force that in addition to genetic drift may explain the divergence of advertisement calls of *P. thaul* along its wide geographical distribution. To accomplish this, we carried out playback experiments with males from three distant populations of this species pertaining to each of the bioacoustic groups found in previous studies and characterized their evoked vocal responses (EVRs) to local and foreign call imitations. The second goal is to study the acoustic parameters to which males of each population respond preferentially, to identify cues that account for responsiveness to local and foreign calls. With this purpose we analyzed the EVRs of males to synthetic calls in which the dominant frequency, pulse rate and modulation depth were systematically modified. If intra-sexual selection plays a role in driving the evolution of acoustic signals of *P. thaul*, we expect that males of each population respond more vigorously to imitations of local relative to foreign calls and also that vocal responses to parametric call variants support the preferences for calls of the different populations. We expect that this exploration will contribute an insight on the evolutionary forces implied in the divergence of the sound communication system in a species with wide geographical distribution.

## Materials and Methods

### Ethical Statements

This study was carried out in private lands with permission from the owners. The procedures employed in this study were approved by the Ethics Committee of the University of Chile (CBA 0423 FMUCH) and comply with regulations for animal care and conservation in Chile (permits 1645 and 7311of the Livestock and Agriculture Service (SAG)).

### Study sites

Playback experiments were carried out for three populations within the distribution range of *P. thaul*: Totoral (northern: 27° 54′ S, 70° 56′ W; N = 18), Los Maitenes (central: 34° 41′ S, 71° 26′ W; N = 18) and Osorno (southern: 40° 35′ S, 73° 03′ W; N = 18). We conducted the experiments in Totoral and Los Maitenes during September and October 2009, and in Osorno during September 2010. At the three sites, frogs produced advertisement calls while perching on emergent low vegetation or floating on the water surface of pools and slow-flowing streams.

### Synthetic stimuli

**Figure 1:**
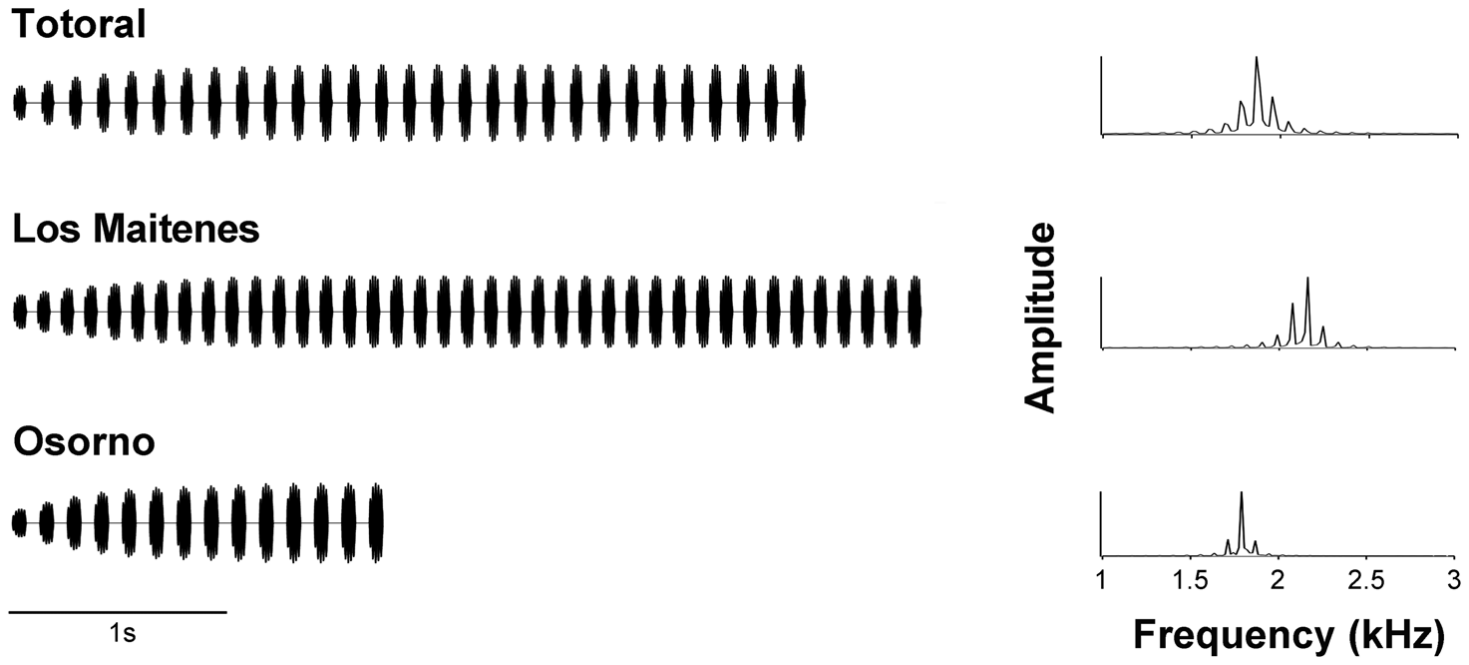
Synthetic stimuli of the Standard call series. Oscillograms (A) and power spectra (B) of stimuli presented to subjects of the three populations.

The structure of the advertisement calls of the three populations included in this study has been characterized in detail previously [29]. We used Adobe Audition 3.0 to generate synthetic stimuli based on averages of the acoustic parameters of the natural calls of the three populations (Fig. 1). Stimuli generated also had rise-time functions that shaped a gradual amplitude increase of their first ten pulses. The parameters for the standard synthetic calls of the three localities, referred to as the Standard call series, are shown in Table 1.

**Table 1:** Acoustic parameters and rise-time functions of the standard synthetic calls for three populations of *Pleurodema thaul*.

	Population		
Acoustic variable	Totoral	Los Maitenes	Osorno
Call duration (s)	3.8	4.3	1.8
Number of pulses	29	39	14
Pulse rate (pulses/s)	8	9	8
Pulse duration (ms)	57	58	65
Inter-pulse interval (ms)	75	53	65
Modulation depth (%)	91	88	58
Dominant frequency (Hz)	1870	2140	1790
Rise-time functions	f(t) = 101.42–59.991 * exp (−2.1926 * t)	f(t) = 101.87–54.358 * exp (−2.3209 * t)	f(t) = 98.915–70.592 * exp (−3.0621 * t)

For an explanation of the rise-time functions see Material and Methods.

**Figure 2:**
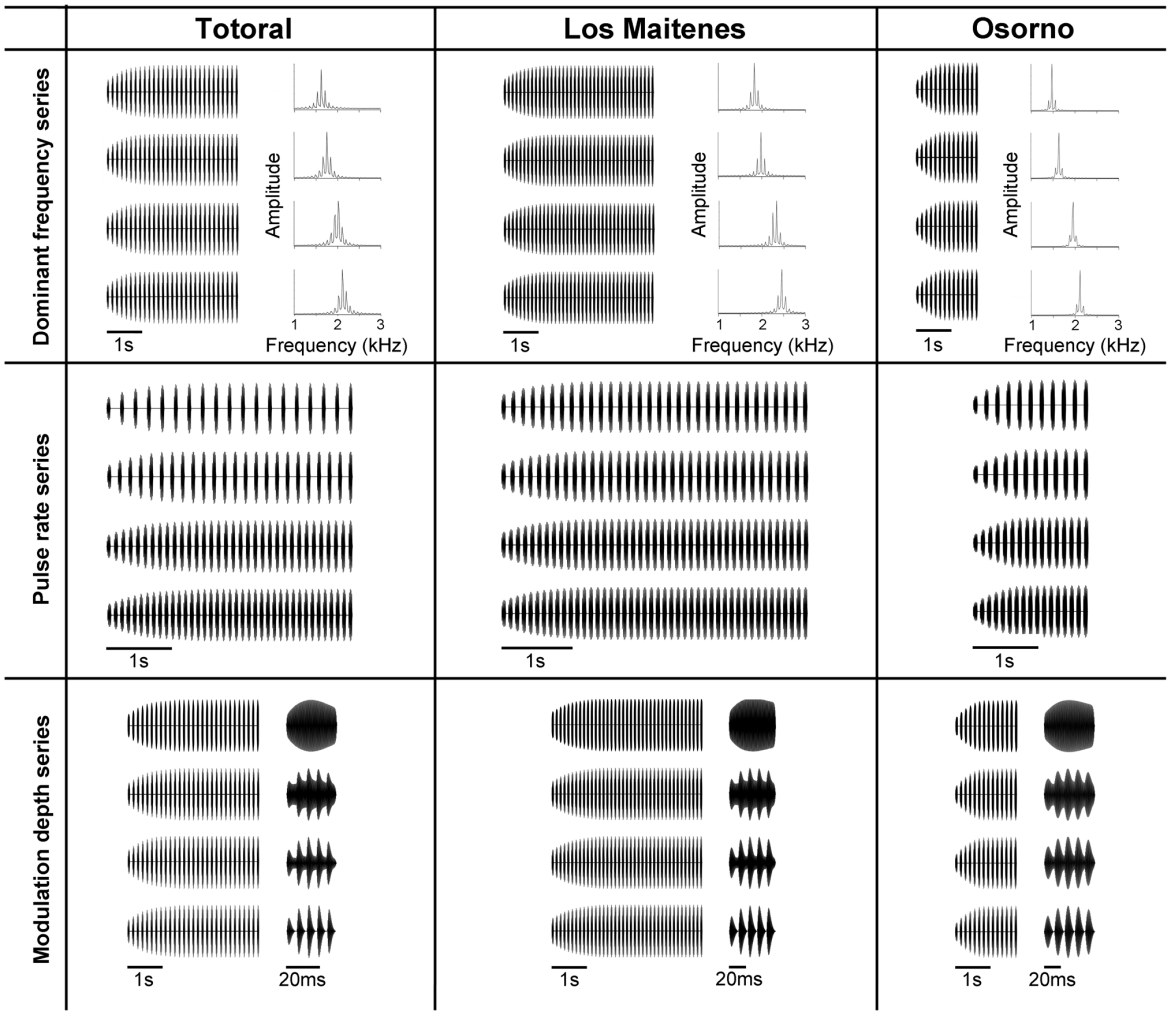
Synthetic stimuli of the Dominant frequency, Pulse rate and Modulation depth series used for each population. For stimuli of the Dominant frequency series, oscillograms of the entire calls and power spectra are shown. For the Pulse rate series only the oscillograms of the entire calls are shown. For stimuli of the Modulation depth series, oscillograms of the entire calls and expanded views of a pulse of each call showing amplitude modulation patterns are shown.

In addition, we generated three series of synthetic calls by modifying three acoustic parameters of the standard synthetic calls of each population. The parameters chosen were those exhibiting a larger variation among populations: dominant frequency, pulse rate and modulation depth [29]. For the Dominant frequency and Pulse rate series, we synthesized four calls having the variable set at one and two standard deviations above and below the average of each population. For the Modulation depth series, arbitrary values of 0, 50, 75 and 100% were used, because the northern and central populations have average modulation depths of 91 and 88%, respectively, and standard deviations would go beyond the 100% upper limit of this variable. The Pulse rate series consisted of stimuli having the same call duration as the standard synthetic call of each population and different number of pulses. For these three series of stimuli, parameters other than the variable manipulated were kept at the same values as for the standard synthetic calls of each population. The parameters used to build the modification of standard synthetic calls for each population are listed in Table 2. All stimuli were saved as WAV files (44.1-kHz sampling rate, 16-bit resolution). These files were used to generate the playback schedules employed in the experiments described below. Figure 2 shows the stimuli comprising the Dominant frequency, Pulse rate and Modulation depth series for the three populations studied.

**Table 2:** Acoustic parameters of the calls of the Dominant frequency, Pulse rate and Modulation depth series for three populations of *Pleurodema thaul*.

Population	Series	−2	−1	Standard calls	+1	+2
Totoral	Dominant frequency (Hz)	1624	1747	1870	1993	2116
	Pulse rate (pulses/s)	5	6	8	9	10
	Modulation depth (%)	0	50	91	75	100
Los Maitenes	Dominant frequency (Hz)	1823	1981	2140	2299	2457
	Pulse rate (pulses/s)	7	8	9	10	11
	Modulation depth (%)	0	50	88	75	100
Osorno	Dominant frequency (Hz)	1472	1631	1790	1949	2108
	Pulse rate (pulses/s)	6	7	8	9	10
	Modulation depth (%)	0	50	58	75	100

−2, −1, +1, +2 indicate standard deviations relative to standard calls for the dominant frequency and pulse rate series.

For the modulation depth series values of 0, 50, 75 and 100% were used because the standard calls of Totoral and Los Maitenes had modulation depth values that did not allow the +1 and +2 variants.

### Experimental settings

For all populations, stimuli were played back with a third generation Ipod Nano (Apple Inc. Cupertino, CA, USA), passed through a custom-made impedance-matched operational amplifier and an attenuator (Hewlett–Packard 355 C/D, Hewlett-Packard, Loveland, CO, USA). The signal was amplified (Alpine 3540, Alpine Electronics of America, Torrance, CA, USA) and broadcast with a loudspeaker (Versatec, 10-cm diameter), which was positioned on a plastic foam floating on the water surface, 1 m from the experimental subjects. The output of the Ipod Nano was connected to the right channel of a digital recorder (Tascam DR-100, Montebello, CA, USA) to record the stimuli during the experiments. To record the EVRs, a directional microphone (Sennheiser ME-66, Electronic GmbH & Co., KG, Wedemark, Germany), connected to the left channel of the digital recorder was positioned 20–40 cm from the experimental subject, pointing to the frog and away from the loudspeaker, to minimize the amplitude of stimuli in the recordings of responses.

Stimuli of the four experimental series were presented in bouts of five repetitions with 6-s intervals between successive repetitions and bouts. The four experimental series were presented sequentially with 60-s intervals in between. At the end of an experiment, a bout of five repetitions of the standard stimulus was presented to control for exposure to prolonged stimulation. By using this schedule, each experimental subject was presented with five bouts of five repetitions of the local standard synthetic call throughout the experiment.

Within each experimental series, stimuli were presented following two presentation orders applied to about half of the animals of each locality. For one group, individuals were stimulated with the Standard call series starting with the local standard synthetic call and following with the two foreign calls presented successively. For the Dominant frequency series and Pulse rate series, the sequence of stimuli was: standard, −2, −1, +1 and +2 standard deviations. For the Modulation depth series, the presentation order was: standard, 0, 50, 75 and 100% amplitude modulation. For the other presentation order, the series of stimuli also started with the local standard synthetic call, but the order of the subsequent stimuli was reversed. This experimental design was chosen to control for effects of the presentation order of the stimuli within a given series.

Before beginning an experiment, the amplitude of the stimuli was set at 70 dB SPL RMS at the position of the experimental subjects by placing the microphone of the sound-level meter (Brüel & Kjaer 2238; Brüel & Kjaer Instruments, Inc., Boston, MA, USA) as close as possible above the subject position (typically 3–5 cm). Neighbors of the experimental subjects were captured or gently disturbed to avoid interferences during the recordings of the EVRs. Recorded EVRs were saved as WAV files (44.1 kHz sampling rate and 16-bit resolution). At the end of each experiment, the focal subject was captured and its SVL (Snout-Vent Length; Traceable Digital Caliper ±0.001 mm) and body weight (Acculab, Pocket Pro balance, Newton, PA, USA; ±0.01 g) measured. In addition, air and water temperature (Digi-Sense 8528-20, Cole-Parmer Instrument Company, Niles, IL, USA) were measured.

### Analysis of EVRs

The recordings obtained were downloaded to a computer and the WAV files thereby generated were filtered (high-pass filter, cutoff: 200 Hz) and analyzed using the Raven Pro 1.3 Software (Laboratory of Ornithology, Cornell University). To analyze the EVRs of the experimental subjects to stimuli we measured four variables: number of pulses (NP), pulse duration (PD), on-off ratio (OR) and dominant frequency (DF). These measurements were applied to time windows which differed depending on the experimental series: for the Standard call series the time window used was 7.8 s, which corresponded to the shortest standard call (Osorno) plus the 6-s inter-stimulus interval. We chose this interval in order to avoid overestimating the calls computed as EVRs after the stimulus onset. For the Dominant frequency, Pulse rate and Modulation depth series, the time windows were the same for a given population: 9.8, 10.3 and 7.8 s for Totoral, Los Maitenes, and Osorno, respectively. These time windows corresponded to the duration of local standard call plus 6-s of the inter-stimulus repetition interval. The responses to the five repetitions of each stimulus were averaged for analysis.

We used multiple regressions to evaluate the effect of the environmental and morphological variables on the four EVR variables (NP, DP, OR, DF) to the local standard call of the Standard call series [38]. In addition, univariate GLMs were applied to compare the environmental and morphological variables (weight, SVL, air and water temperature) among populations (see [39] for GLM analysis). For these analyses, air and water temperatures, weight and SVL were included as independent variables whereas NP, DP, OR and DF as dependent variables. For the Standard call series, EVRs were standardized by dividing the value of an EVR variable to foreign standard calls by the value of the variable in the responses to the local standard call of each population. For the Dominant frequency, Pulse rate and Modulation depth series, the EVRs to the four synthetic variants were divided by the responses to local standard call of each series. We chose this standardization mode because each subject had a particular response pattern with different levels of vocal activity throughout the experiment. Subsequently, GLMs (General Linear Models) were applied to analyze the different variables as follows: first, to study the variation of NP, DP, OR and DF within each experimental series we used repeated measures GLMs. Second, the four EVR variables in response to the five presentations of the standard synthetic call were compared with repeated measures GLMs to assess the variation of the vocal activity throughout the experimental session. All multiple comparisons were performed with Tukey's tests. All statistical analyses were performed with the STATISTICA 8.0 software (StatSoft, Inc. Tulsa, Oklahoma, USA).

## Results

### Environmental and morphological variables

**Figure 3:**
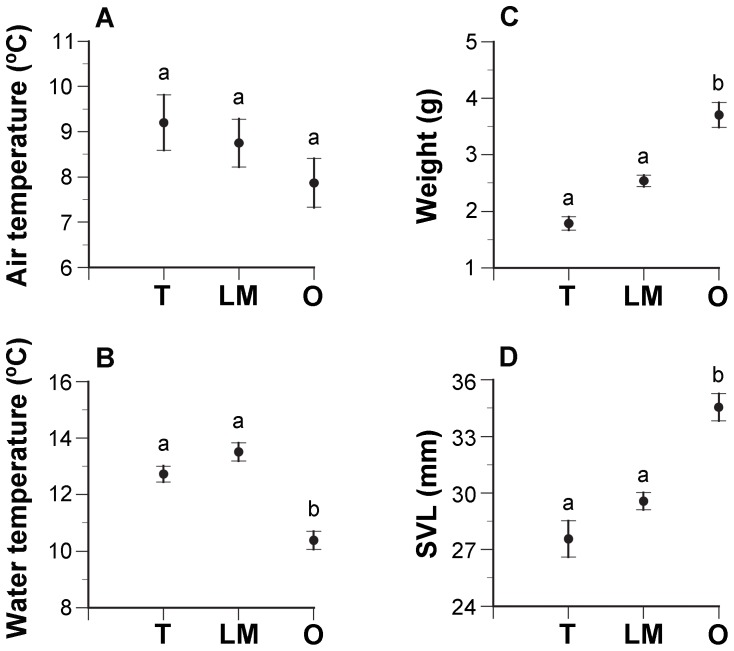
Environmental and body variables measured for recorded subjects from the three populations studied. A: air temperature B: water temperature, and C: body weight, B: snout-vent length. Filled circles and bars represent averages and standard errors, respectively. Different lowercase letters indicate significant differences among populations. Abbreviations: T: Totoral, LM: Los Maitenes, O: Osorno.

Air temperatures measured after recording the vocal responses of each individual did not differ among populations (*F*_2,51_ = 1.4585, *p* = 0.2421; Fig. 3A), however water temperature showed significant differences among populations (*F*_2,51_ = 28.118, *p*<0.0001). Water temperatures in Totoral and Los Maitenes were higher than in the Osorno population. (Tukey's test: *p*<0.05, for both comparisons; Fig. 3B). In addition, body weight of the subjects captured after the recordings showed significant differences among populations (*F*_2,39_ = 33.443, *p*<0.0001). Frogs from Osorno had larger body weights than those from Los Maitenes and Totoral (Tukey's test: *p*<0.05, for both comparisons; Fig. 3C). SVLs also showed significant differences among populations (*F*_2,39_ = 25.351, *p*<0.0001); frogs from Osorno had larger SVLs than those from Los Maitenes and Totoral (Tukey's test: *p*<0.05, for both comparisons; Fig. 3D). Multiple regressions showed no dependence of the EVR variables measured (NP, DP, OR and DF) on temperatures or body dimensions (*p*>0.05), with the only exception of a positive relationship between body weight and pulse duration in Los Maitenes (Multiple regression: *F*_4,9_ = 4.3393, *p* = 0.0448).

### Vocal activity throughout the experiment

EVR variables of males of *P. thaul* between the two presentation orders of stimuli were similar in most cases. Only 18 out of 228 comparisons carried out for all EVR variables in all experimental series showed significant differences between the two orders (Totoral, Modulation depth series: 0%: NP, 0%: PD, 0%: OR, 75%: DF, 100%: DF; Los Maitenes, Pulse rate series: LM: NP, Modulation depth series: LM: NP, 0%: NP, LM: OR, 0%: OR, final control: NP, OR; Osorno: Dominant frequency series: Osorno standard call: PD, 1.631 kHz: PD, Pulse rate series: Osorno standard call: NP, Osorno standard call: PD, Osorno standard call: OR, Modulation depth series: Osorno standard call: PD, p<0.0479 for all comparisons). Because results for most of the comparisons were similar, the data for both presentation orders were pooled for further analyses.

EVRs to the presentations of local standard calls throughout the experiment within each population were similar in Totoral and Osorno. In Totoral, all variables measured were similar among the EVRs to the five bouts of repetitions of the local standard call presented throughout the experiment (NP: *F*_4,60_ = 1.1837, *p* = 0.3272; PD: *F*_4,48_ = 0.9855, *p* = 0.4244; OR: *F*_4,60_ = 1.2186, *p* = 0.3125; DF: *F*_4,48_ = 0.6184, *p* = 0.6515). Similarly, in Osorno all variables were similar among the five bouts of repetitions of local standard call presented throughout the experiment (NP: *F*_4,44_ = 0.6382, *p* = 0.6380; PD: *F*_4,40_ = 2.3179, *p* = 0.0737; OR: *F*_4,44_ = 0.7601, *p* = 0.5565; DF: *F*_4,40_ = 0.9804, *p* = 0.4291). In Los Maitenes, NP in response to the first bout of repetitions of the local standard call was lower than in response to the third bout of this stimulus (*F*_4,60_ = 2.6636, *p* = 0.0409; Tukey's test: *p* = 0.0394), PD was shorter in response to the first than to both the third and fifth bouts of the local standard call (*F*_4,56_ = 4.1045, *p* = 0.0055; Tukey's test: first-third presentation, *p* = 0.0181, first-fifth presentation, *p* = 0.0040). Also OR was lower in responses to the first as compared to the third and fifth presentation of the local standard call (*F*_4,60_ = 3.1289, *p* = 0.0210; Tukey's test: first-third presentation, *p* = 0.0332, first-fifth presentation, *p* = 0.0333). DF was similar among all presentations of the local standard synthetic call for this population (*F*_4,56_ = 0.7970, *p* = 0.5322) (Figs. S1, S2 and S3).

### Responses to the four experimental series

EVRs of males gave different responses to stimuli within each of the four experimental series. The response patterns of males from Totoral and Los Maitenes were similar; both populations responded in the Standard call series with higher NP and OR to the standard calls of these populations, relative to the standard call of Osorno (Table 3 and 4; Figs. S1 and S2). In response to the Modulation depth series, both populations responded with lower NP and OR to the stimulus lacking amplitude modulation (0%) relative to the standard synthetic call, however only in the population of Totoral the differences reached significant levels (Table 3 and 4; Figs. S1 and S2). Males of these two populations in general gave similar responses to all stimuli of the Dominant frequency and Pulse rate series. However, differences occurred for some of the EVR variables measured, as follows: males from Totoral responded with calls having shorter pulses to the 1.624 kHz relative to the 1.993 kHz stimulus of the Dominant frequency series and with calls having lower DF to the standard call relative to the 6 pulses/s stimulus of the Pulse rate series (Table 3; Fig. S1). Finally, males from Los Maitenes responded with calls having shorter pulses to the 50% stimulus relative to the 0 and 100% stimuli in the Modulation depth series (Table 4; Fig. S2). EVRs of males from Osorno had a general pattern different from the populations of Totoral and Los Maitenes: they responded in the Standard call series to the local standard call with lower values for the four variables relative to the two foreign standard calls. EVRs as measured by NP and OR were significantly lower to the standard call of Osorno relative to the standard call of Totoral, whereas PD to the standard call of Los Maitenes was significantly higher than to the standard call of Totoral (Table 5; Fig. S3). In general, males of Osorno did not show significant differences in their EVRs to the three other series of stimuli, with the exception of the Dominant frequency series in which PD was longer in response to the 1.631 kHz stimulus relative to the standard local call. In addition, males from Osorno responded in the Pulse rate series with higher DF to the 6 pulses/s stimulus relative to the standard local call (Table 5; Fig. S3).

**Table 3:** Repeated measures GLMs and Tukey's tests for EVR variables (NP, DP, OR and DF) within four series of stimuli presented to males from the Totoral population of *P. thaul*.

EVR variable	Stimuli series	df	F	P	Tukey's tests
NP	Populations	2,34	5.37	**0.0094**	TvsO; LMvsO
	Dominant frequency	4,68	1.58	0.1902	
	Pulse rate	4,64	0.24	0.9143	
	Modulation depth	4,60	4.44	**0.0033**	Tvs0
PD	Populations	2,26	3.47	**0.0460**	
	Dominant frequency	4,60	3.12	**0.0213**	1993vs1624
	Pulse rate	4,56	0.27	0.8946	
	Modulation depth	4,40	0.94	0.4532	
OR	Populations	2,34	5.87	**0.0065**	TvsO; LMvsO
	Dominant frequency	4,68	1.70	0.1608	
	Pulse rate	4,64	0.20	0.9356	
	Modulation depth	4,60	3.65	**0.0099**	Tvs0
DF	Populations	2,26	0.10	0.9029	
	Dominant frequency	4,56	0.68	0.6103	
	Pulse rate	4,52	4.05	**0.0062**	6vsT
	Modulation depth	4,40	0.24	0.9155	

In the last column, multiple comparisons showing significant differences are listed. The significance level was 0.05.

Abbreviations: NP: number of pulses, PD: pulse duration, OR: on-off ratio, DF: dominant frequency. In the Tukey's test column T, LM and O refer to standard calls for Totoral, Los Maitenes and Osorno, respectively. In this column, letters and numbers separated by “vs” indicate the stimuli variables compared showing significant differences.

**Table 4:** Repeated measures GLMs and Tukey's test for EVR variables (NP, DP, OR and DF) within four series of stimuli presented to males from the Los Maitenes population of *P. thaul*.

EVR variable	Stimuli series	df	F	P	Tukey's test
NP	Populations	2,34	11.36	**0.0002**	LMvsO; TvsO
	Dominant frequency	4,68	0.8227	0.5152	
	Pulse rate	4,68	1.06	0.3824	
	Modulation depth	4,68	1.21	0.3164	
PD	Populations	2,16	0.73	0.4965	
	Dominant frequency	4,36	0.64	0.6367	
	Pulse rate	4,44	2.32	0.0717	
	Modulation depth	4,20	3.35	**0.0296**	0vs50; 100vs0
OR	Populations	2,34	10.42	**0.0003**	LMvsO; TvsO
	Dominant frequency	4,68	0.66	0.6221	
	Pulse rate	4,68	1.06	0.3824	
	Modulation depth	4,68	1.06	0.3854	
DF	Populations	2,14	1.51	0.2555	
	Dominant frequency	4,36	0.63	0.6480	
	Pulse rate	4,44	1.54	0.2064	
	Modulation depth	4,20	0.03	0.9981	

In the last column, the multiple comparisons showing significant differences are listed. The significance level was 0.05. Abbreviations and symbols as in Table 3.

**Table 5:** Repeated measures GLMs and Tukey's test for EVRs variables (NP, DP, OR and DF) within four series of stimuli presented to males from the Osorno population of *P. thaul*.

EVR variable	Stimuli series	df	F	P	Tukey's test
NP	Populations	2,34	5.07	**0.0119**	TvsO
	Dominant frequency	4,64	1.74	0.1517	
	Pulse rate	4,64	2.44	0.0558	
	Modulation depth	4,64	1.78	0.1411	
PD	Populations	2,34	5.07	**0.0119**	TvsO; LMvsT
	Dominant frequency	4,64	1.74	**0.1517**	1631vsO
	Pulse rate	4,64	2.44	0.0558	
	Modulation depth	4,64	1.78	0.1411	
OR	Populations	2,34	5.07	**0.0119**	TvsO
	Dominant frequency	4,64	1.74	0.1517	
	Pulse rate	4,64	2.44	0.0558	
	Modulation depth	4,64	1.78	0.1411	
DF	Populations	2,34	5.07	0.0119	
	Dominant frequency	4,64	1.74	0.1517	
	Pulse rate	4,64	2.44	**0.0558**	6vsO
	Modulation depth	4,64	1.78	0.1411	

In the last column, the multiple comparisons showing significant differences are listed. The significance level was 0.05. Abbreviations and symbols as in Table 3.

## Discussion

Males of *P. thaul* of the three populations studied show different EVR patterns to standard synthetic calls. Interestingly, the responses show a general correspondence with the variation of the natural advertisement calls among these localities: the northern and central populations, having similar calls between them and diverging from the southern population [29], also have similar response patterns and both differ in signal structure and response pattern from the southern population. When presented with the local standard synthetic call, males from Totoral and Los Maitenes gave strong EVRs, with higher number of pulses and on-off ratio relative to their responses to the standard synthetic call of Osorno. In addition, males from Totoral and Los Maitenes gave strong vocal responses to the standard synthetic call of each other population as well. Similar responses for stimuli having a structure resembling own calls have been reported in comparisons among different species [40] [41] and in studies using stimuli for which different parameters are varied systematically [42]. In remarkable contrast, males of the Osorno population responded with higher number of pulses and on-off ratio to the standard call of Totoral, relative to the local standard stimulus.

The patterns of responsiveness to foreign standard calls by males of the populations of *P. thaul* analyzed are related to selectivities unveiled by the series of stimuli in which call features were varied systematically. For instance, the stronger responses of males of Osorno to the standard calls of Totoral and Los Maitenes (e.g. higher number of pulses and on-off ratio) could be related to the higher number of pulses of these stimuli relative to the Osorno standard call, since the responses of the southern population to the Pulse rate series showed marginal differences between stimuli (Table 5, p = 0.0588), suggesting that there is a direct relationship between the number of pulses of stimuli and the number of pulses in their EVRs. In contrast, the lower responses of males of Totoral and Los Maitenes to the Osorno standard call is not likely to depend on the lower number of pulses of this signal, since subjects from the northern and central localities did not change their vocal activity in response to the stimuli with different number of pulses composing the Pulse rate series. In addition, responses to the Modulation depth series also bear a relationship with the amplitude modulation patterns characteristic of the different populations. Males of Totoral and Los Maitenes gave low responses to stimuli lacking amplitude modulation and also to the Osorno standard call, which had modulation depths well below the patterns characteristic of northern and central populations. In contrast, males of Osorno responded strongly to stimuli throughout the entire modulation depth range and also to the standard calls of Totoral and Los Maitenes, which had modulation depths well above the local standard call. A neurophysiological study has revealed a high selectivity of midbrain auditory neurons of *P. thaul* for temporal patterns of conspecific calls, in particular for pulse rate, which provides support for the preferences in the evoked vocal responses unveiled in the current behavioral study [43].

The similarities in the EVRs between males from Totoral and Los Maitenes, and their differences with those of males from Osorno are in agreement with the pattern of geographic variation of the advertisement call of this anuran. Calls of the northern and central populations are very similar in their temporal characteristics, diverging clearly from the southern population, the most important differences residing in the modulation depth [29]. Overall, EVR patterns and natural geographical variation in the temporal structure of calls are highly congruent in *P. thaul*.

Our results show that the remarkable geographic variation in the EVRs of males of *P. thaul* is also related to their selectivity for particular acoustic variables, as discussed above. This would indicate that males from different populations along the distribution range of *P. thaul* have distinct strategies of response to familiar and unfamiliar signals. The stronger responses of males from northern and central populations to stimuli similar to those from their own populations are likely due to the significance of these sounds as potential direct competitors for territories or access to females. A similar condition has been described in male *Geospiza* finches which show strong vocal responses to the local call types as compared to calls of conspecific males from foreign islands [44] [45] [46]. Preferences in vocal responses of this kind but for conspecific relative to heterospecific signals have also been shown to occur in anurans [40] [41].

In contrast with the northern and central populations, the strong vocal responses exhibited by males of the southern group to stimuli from foreign populations which contain a higher number of pulses than the local stimulus could represent an example of responses of “supernormal” stimuli, a behavioral phenomenon widely spread in vertebrates [47] [48] [49]. Overall, the EVR patterns found in this study underline the relevance of interactions among males for the evolution of signals and sound communication systems as has been proposed to occur in other animals [50] [51] [52] [53] [54] [55] [56].

Variation in responsiveness to stimuli of different structure among populations of *P. thaul* sheds evidence on the operation of intra-sexual selection processes in the inter-population call divergence observed. Males of the three populations of *P. thaul* studied respond with a similar general pattern to acoustic stimulation, but differ in their sensitivities to particular stimuli. Namely, males of all populations give stronger vocal displays as measured in the number of pulses or on-off ratio to variants of the parameters to which they are particularly sensitive (i.e. northern and central populations are remarkably sensitive to variations in the modulation depth whereas the southern population is particularly sensitive to changes in the number of pulses). By increasing their responsiveness males enhance their detectability in the chorus aggregation, thereby improving their chances to get a mate. This process in which the differential reproductive success results from differential success in social competition has been referred to as social selection, and results in a rapid exaggeration of the relevant traits [11] [53]. The remarkable divergence in the vocal responses of the populations of *P. thaul* could have been established on the course of prolonged inter-male interactions within populations. In this species, males form chorusing aggregations maintained over night time, in which individuals are persistently exposed to vocal agonistic interactions with other males, resulting in the establishment of territories and female attraction [29] [57]. In this challenging context, intra-sexual selection may promote by itself the evolution of signals emitted in each population without implying an active participation of inter-sexual selection. The process by which females are directed towards males simply by exaggerated advertisement of signal parameters has been termed passive attraction [11] [58] [59].

The results reported render *P. thaul* an attractive model of study, with an extensive geographic variation in its acoustic signals in correspondence with diverse male response strategies related to the divergence of the sound communication system among populations. It would be particularly relevant to determine if the geographic variation in both signal structure and responses strategies in *P. thaul* is in addition accompanied by differences in female preferences. Ongoing studies of female phonotactic responses would allow us to assess the relative importance of intra- and inter-sexual selection for reproductive isolation and eventual speciation processes affecting this taxon.

## Supporting Information

Figure S1 EVR variables measured for males from Totoral to four stimuli series. Name of the stimuli series (Standard, Dominant frequency, Pulse rate and Modulation depth) are indicated at the bottom of the figure. The values of each variant within a stimulation series are shown in the abscissa. Stimuli order corresponds to presentation order 1 (see text). Abbreviations: T, LM and O: standard calls of Totoral, Los Maitenes and Osorno, respectively. Filled circles and bars represent averages and standard errors, respectively.
(TIF)

Figure S2 EVR variables measured for males from Los Maitenes to four stimuli series. Name of the stimuli series (Standard, Dominant frequency, Pulse rate and Modulation depth) are indicated at the bottom of the figure. The values of each variant within a stimulation series are shown in the abscissa. Stimuli order corresponds to presentation order 1 (see text). Abbreviations: T, LM and O: standard calls of Totoral, Los Maitenes and Osorno, respectively. Filled circles and bars represent averages and standard errors, respectively.
(TIF)

Figure S3 EVR variables measured for males from Osorno to four stimuli series. Name of the stimuli series (Standard, Dominant frequency, Pulse rate and Modulation depth) are indicated at the bottom of the figure. The values of each variant within a stimulation series are shown in the abscissa. Stimuli order corresponds to presentation order 1 (see text). Abbreviations: T, LM and O: standard calls of Totoral, Los Maitenes and Osorno, respectively. Filled circles and bars represent averages and standard errors, respectively.
(TIF)
