# Supplementary figures and images for: Divergence of Acoustic Signals in a Widely Distributed Frog: Relevance of Inter-Male Interactions
Source: PLoS One. 2014 Jan 28;9(1):e87732. doi: 10.1371/journal.pone.0087732 (PMC3905042; doi:10.1371/journal.pone.0087732)

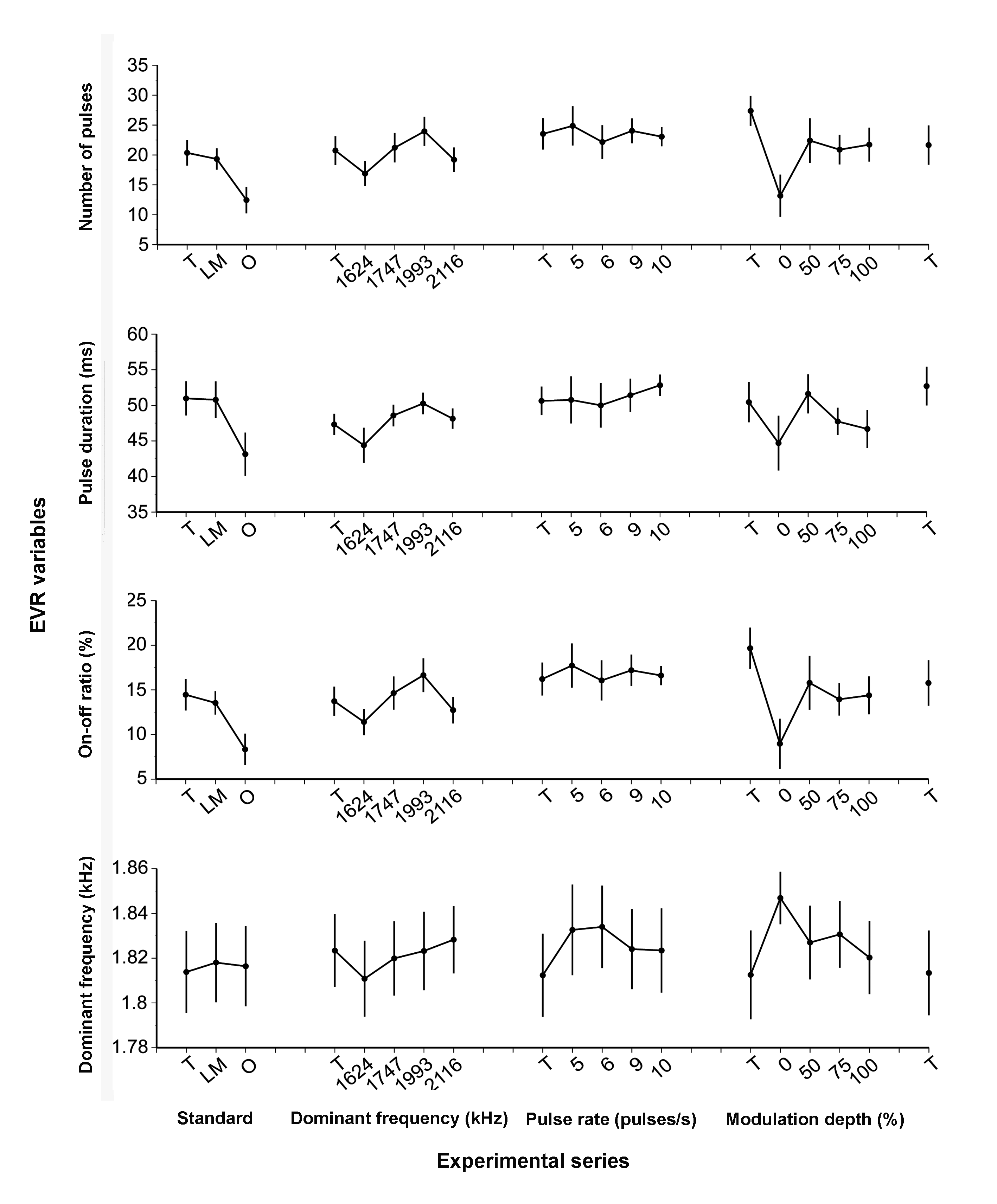

Supplement: Figure S1 — EVR variables measured for males from Totoral to four stimuli series. Name of the stimuli series (Standard, Dominant frequency, Pulse rate and Modulation depth) are indicated at the bottom of the figure. The values of each variant within a stimulation series are shown in the abscissa. Stimuli order corresponds to presentation order 1 (see text). Abbreviations: T, LM and O: standard calls of Totoral, Los Maitenes and Osorno, respectively. Filled circles and bars represent averages and standard errors, respectively. (TIF) [file pone.0087732.s001.tif]

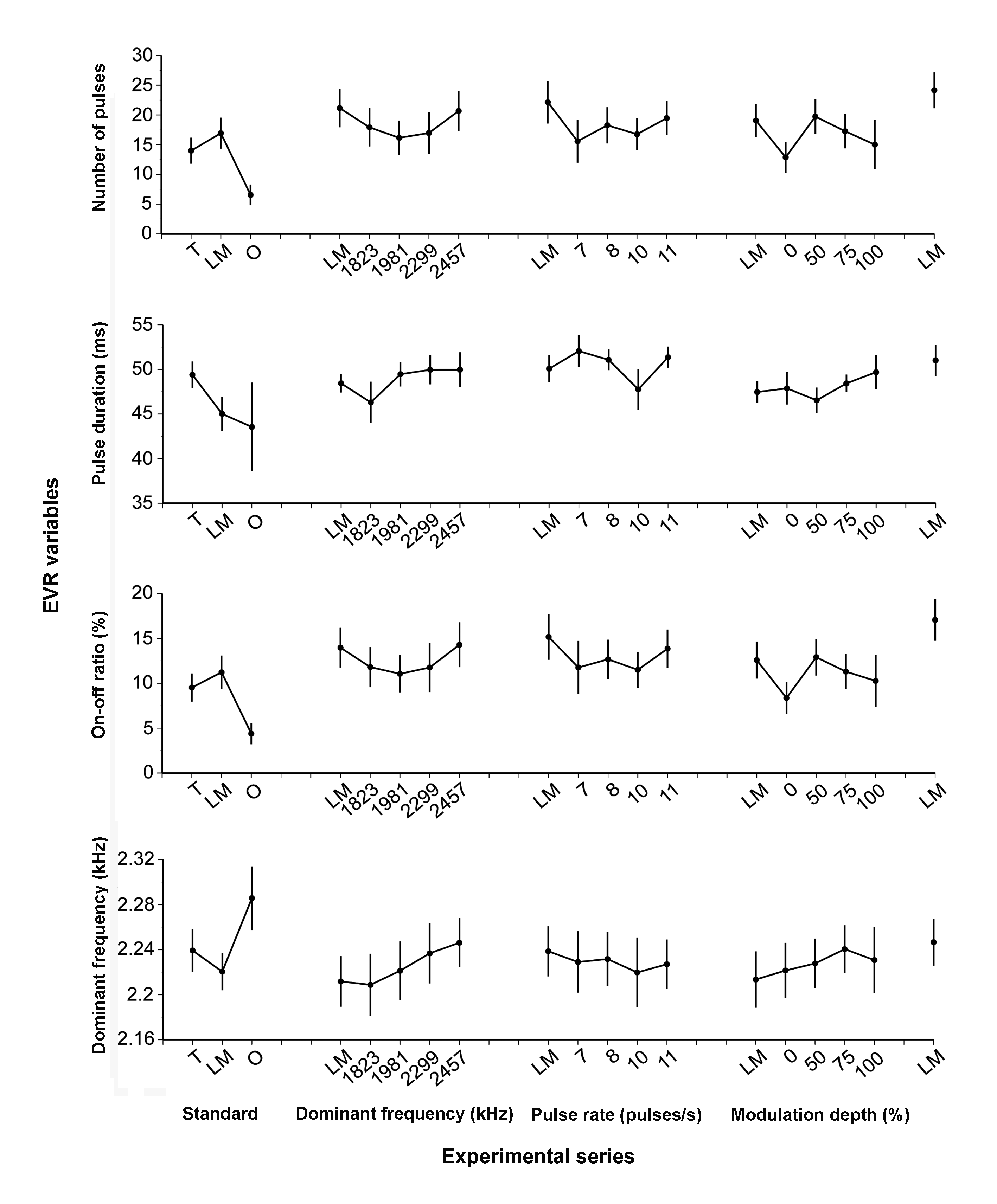

Supplement: Figure S2 — EVR variables measured for males from Los Maitenes to four stimuli series. Name of the stimuli series (Standard, Dominant frequency, Pulse rate and Modulation depth) are indicated at the bottom of the figure. The values of each variant within a stimulation series are shown in the abscissa. Stimuli order corresponds to presentation order 1 (see text). Abbreviations: T, LM and O: standard calls of Totoral, Los Maitenes and Osorno, respectively. Filled circles and bars represent averages and standard errors, respectively. (TIF) [file pone.0087732.s002.tif]

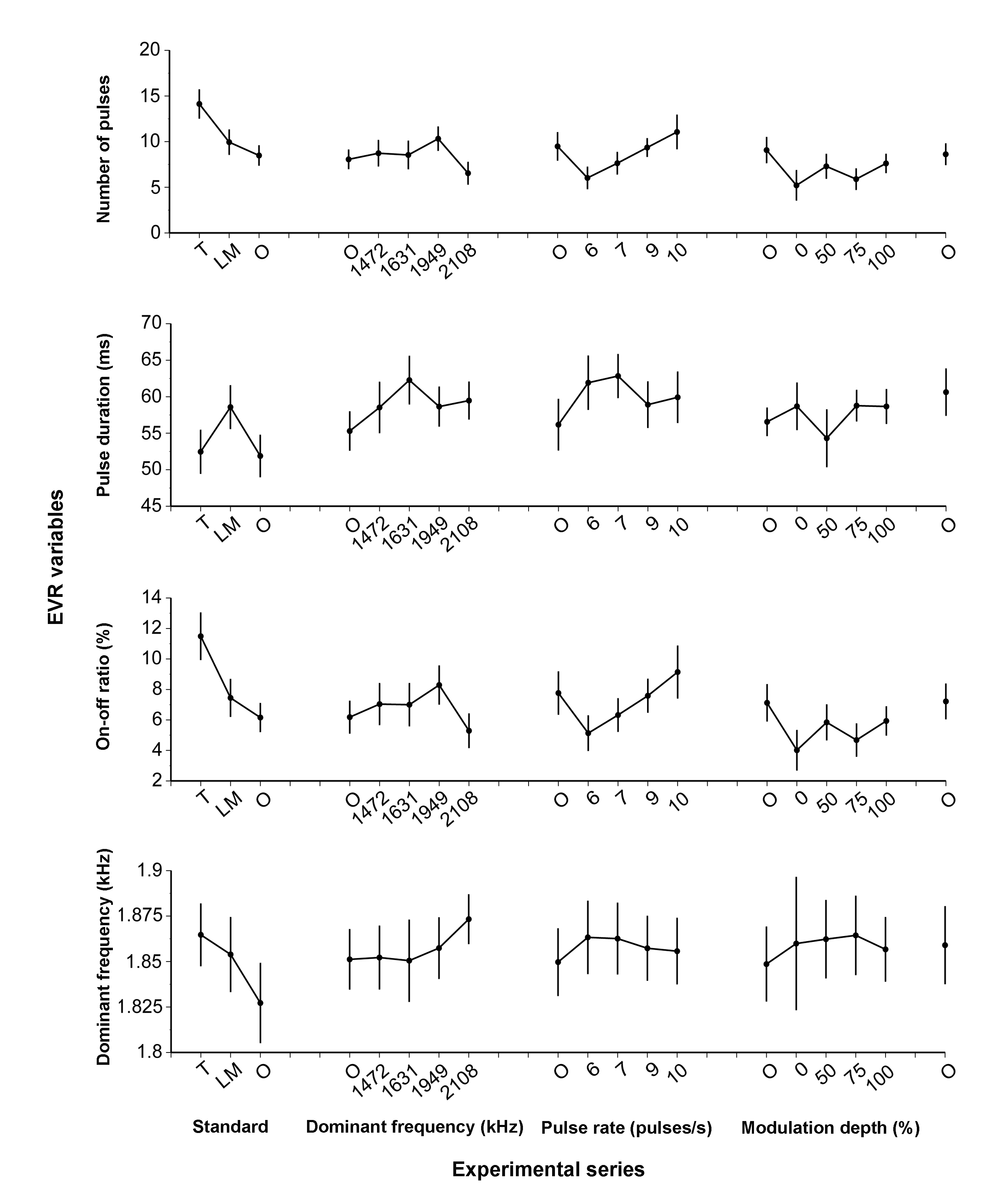

Supplement: Figure S3 — EVR variables measured for males from Osorno to four stimuli series. Name of the stimuli series (Standard, Dominant frequency, Pulse rate and Modulation depth) are indicated at the bottom of the figure. The values of each variant within a stimulation series are shown in the abscissa. Stimuli order corresponds to presentation order 1 (see text). Abbreviations: T, LM and O: standard calls of Totoral, Los Maitenes and Osorno, respectively. Filled circles and bars represent averages and standard errors, respectively. (TIF) [file pone.0087732.s003.tif]
